# Transient patulous eustachian tube in severe anorexia nervosa: A prospective observational study

**DOI:** 10.1002/lio2.846

**Published:** 2022-07-04

**Authors:** Scott E. Mann, Jeff Hollis, Trudy Frederics, Ashlie Watters, Judy Oakes, Stephen P. Cass, Philip S. Mehler

**Affiliations:** ^1^ Department of Otolaryngology Head and Neck Surgery University of Colorado School of Medicine Aurora Colorado USA; ^2^ Department of Surgery Denver Health Hospital Authority Denver Colorado USA; ^3^ Department of Medicine Denver Health Hospital Authority Denver Colorado USA; ^4^ Department of Medicine University of Colorado School of Medicine Aurora Colorado USA; ^5^ ACUTE Center for Eating Disorders Denver Health and Hospital Authority Denver Colorado USA; ^6^ Audiology Services Denver Health Hospital Authority Denver Colorado USA; ^7^ Eating Recovery Center Denver Colorado USA

**Keywords:** anorexia nervosa, autophony, Eustachian tube dysfunction, patulous eustachian tube

## Abstract

**Objectives:**

To understand the presence of transient autophony symptoms in patients being treated for severe anorexia nervosa (AN), and whether those symptoms were due to patulous eustachian tube (PET).

**Methods:**

A prospective observational study was performed in patients requiring admission for treatment of severe AN. All enrolled patients completed The Eustachian Tube Dysfunction Questionnaire (ETDQ‐7) and were screened for symptoms of autophony. If patients reported autophony and had a score of ≥14.5 on the ETDQ‐7 they were asked to undergo comprehensive audiological testing and an evaluation with an otolaryngologist.

**Results:**

Of the 73 patients enrolled in the study, 35 patients (44%) reported autophony and 36 (49%) scored 14.5 or higher on the ETDQ‐7. Of the 16 (22%) patients who had both autophony and an ETDQ‐7 score of 14.5 or higher, 7 patient s (representing 11 symptomatic ears) underwent evaluations by audiology and otolaryngology. Every evaluation of a symptomatic ear revealed objective evidence of PET. Nine of 11 (81.8%) symptomatic ears had subjectively resolved within 12 days of admission after nutritional rehabilitation and weight gain.

**Conclusion:**

Transient autophony in severe AN patients is due to PET, and was present in at least 8% of patients within our cohort. Further study is warranted to understand the quality of life impact and pathophysiology of transient PET in this patient population.

## Graphical abstract

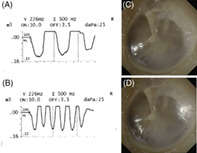

## 1 INTRODUCTION

Patulous eustachian tube (PET) is a type of eustachian tube dysfunction characterized by an abnormally patent tube and prolonged communication between the middle ear and nasopharynx. It can be a bothersome chronic condition and has no known consistently reliable treatment. Symptoms of PET include aural fullness, subjective hearing loss, and autophony.^1^ Autophony is the hyperperception of one's own voice or breath sounds. Though autophony can be caused by several conditions, it is often considered the hallmark symptom of PET and has been included as an essential feature in diagnostic frameworks for PET.^2^, ^3^

Weight loss is a frequently reported risk factor for PET. Several studies show an increased incidence of PET following rapid weight loss after bariatric surgery,^4^, ^5^ and PET occurring following weight loss from other causes such as chronic diseases or cancer therapy is also described.^6^, ^7^ It has been postulated that loss of adipose tissue surrounding the cartilaginous portion of the ET plays a causal role in the development of PET.^2^ However, there are instances when PET occurs during periods of weight gain such as pregnancy.^8^ Despite weight loss being identified as an important risk factor in the development of PET, there are no studies that demonstrate reversal of PET with weight gain.

Anorexia Nervosa (AN) has also been reported as a risk factor to development of PET.^6^ AN is an eating disorder characterized by abnormally low‐body weight, distorted perception of body weight, and a fear of gaining weight. Patients with AN may undergo periods of rapid weight loss due to their disordered eating. Though there have been case reports of PET symptoms developing after rapid weight loss due to AN, this has not been studied systematically.^9^, ^10^, ^11^ Godbole and Key reported a series of three cases of autophony in AN, and noted that the symptoms had tremendous psychological impact, impairing their ability to participate in treatment and worsening a preoccupation with somatic symptoms.^11^ Karwautz et al. reported a single case of severe AN with autophony that was presumed to be from PET. In this case the patient was similarly distressed, but autophony resolved within 2 days of admission.^10^ Despite limited reports in the literature, autophony in severe AN patients is common. In a cross‐sectional survey study of otologic symptoms in 101 patients admitted for treatment of a severe eating disorder, 43% of patients reported autophony within the prior 24 h.^12^

Anecdotally, it is common for patients admitted for treatment of severe AN to report an intrusive autophony that then resolves within days of an admission to medically stabilize and begin weight restoration. This is intriguing given that PET is often a chronic condition and would not be expected to resolve until after significant restoration of body weight. The questions arise whether the transient autophony, as also reported by Karwautz et al. is really due to PET, and if it is, whether some factor other than weight gain is responsible for the rapid improvement of symptoms. Importantly, none of the previously reported cases included objective evaluations for PET. Diagnosis of PET requires objective evidence such as respiratory induced tympanic membrane (TM) oscillations during binocular microscopy or reflex decay testing.^6^, ^13^

We hypothesize that the transient autophony reported by patients diagnosed with severe AN is, in fact, caused by PET, and that detailed investigation of the acute nature and rapid resolution may provide new insights into the pathogenesis of PET. We conducted a prospective observational study of patients with severe AN including objective testing of eustachian tube function in patients with autophony. The primary aim was to determine which patients experienced autophony and whether this symptom was due to PET.

## 2 MATERIALS AND METHODS

A prospective observational study was conducted to investigate autophony symptoms in patients receiving in‐patient treatment for severe eating disorders. Newly admitted patients at the ACUTE center for Eating Disorders at Denver Health Medical Center, from February 2019 to March 2020, were invited to participate. Patients were eligible for inclusion in the study if their admission % ideal body weight (%IBW) was <80%, if they were available to participate within the first 4 days of admission, and if they were diagnosed with AN restricting subtype (AN‐R), AN binge‐purge subtype (AN‐BP) or avoidant restrictive intake disorder (ARFID) per the DSM V criteria on admission.^14^ Prior to enrollment, potential participants were given comprehensive information about the study purpose, design, and hypotheses. Participation in each portion of the study was voluntary and the study was reviewed and approved by the Colorado Multiple Institutional Review Board.

### 2.1 Screening for autophony

Enrolled patients were asked to complete the 7‐Item Eustachian Tube Dysfunction Patient Questionnaire (ETDQ‐7), a validated clinical tool for assessment of eustachian tube dysfunction.^15^ The ETDQ‐7 measures the severity of seven different symptoms of ETD, during the past month, on a scale from 1–7, with 1–2 being “No Problem” to 6–7 being “Severe Problem”. A score of 14.5 or greater has been shown to be correlated with clinically significant eustachian tube disorder.^15^ The ETDQ‐7 identifies patients with either dilatory or patulous ET dysfunction but does not differentiate between the two.^13^, ^16^, ^17^ Because the ETDQ‐7 does not assess autophony, patients were also asked three screening questions about autophony symptoms. These questions were taken from a screening questionnaire developed for a prior study of otologic symptoms in eating disorder patients.^12^

In the past 24 h have you experienced any of the following symptoms?

- An echoing sound in your head
- Abnormally loud sound of your own voice
- Abnormally loud sound of your own breathing, chewing, or swallowing

### 2.2 Otologic evaluation

If patient answered “Yes” to any of the three autophony questions, and if responses to the ETDQ‐7 resulted in a score of 14.5 or greater, they were determined to have clinically significant ear symptoms with autophony, suggesting PET. These patients were asked to undergo further objective testing including audiological evaluations and a consultation with an otolaryngologist. Only patients who volunteered to undergo additional testing, were medically stable enough for temporary transfer to the Otolaryngology and Audiology clinics, and who reported symptoms that were active at time of enrollment, were included in the testing group.

Audiological testing was performed within a sound‐proof booth and included pure tone air conduction threshold testing, tympanometry, acoustic reflexes with reflex decay. To test for respiratory oscillations of TM, reflex decay testing was performed during patient breathing with closed mouth and occluded contralateral nostril. This was performed with slow and fast rates for each patient. Oscillations of reflex decay that change with the rate of respiration were determined to be respiratory TM excursions.

The evaluation by the otolaryngologist included an otologic‐focused history and complete head and neck physical exam. Binocular otomicroscopy was performed in both upright and supine positions on all patients. Additional video otoscopy was performed in some patients.

Any patient with repeatable rate‐dependent respiratory oscillations during reflex decay testing was determined to have PET. Similarly, any patient with observed (repeatable and rate dependent) respiratory excursions of the TM during otomicroscopy or video otoscopy was determined to have PET.

Patient demographics, including age in years and sex (male or female), along with anthropometric measurements (weight in kilograms, height in meters) and nursing assessments were obtained from chart review. Lipodystrophy analysis/whole body scans were performed using the Hologic QDR Series model Discovery‐W. Duration of eating disorder disease was self‐reported and recorded on admission. BMI was calculated using the formula: weight (kg)/height (m)^2^ and %IBW was calculated using the Hamwi method.^18^ Data collected from the electronic medical record (Epic) was stored on a RedCap database.

The size of the study was determined by the time period of data collection. Patients were enrolled starting in February 2019 and the study was ended when COVID‐19 related restrictions suspended clinical research activities in March 2020.

### 2.3 Statistical analysis

Univariate statistics were used to describe the cohort. Shapiro‐Wilks test was used to determine the distribution of continuous variables. Based on the distribution, patient characteristics, used to describe the severity of ETD, were ascertained with independent sample t‐tests. When appropriate, degrees of freedom are shown in parentheses after the test statistic. *p* values of <.05 were considered statistically significant, and all analyses were completed using SAS Enterprise Guide software version 7.1 (SAS Institute, Cary, NC).

## 3 RESULTS

Three hundred and two new admissions were screened and for inclusion in the study. Of these, 182 did not meet all inclusion criteria, 30 declined to participate, and 17 were repeat admissions that had previously participated. In total, 73 patients consented to participate and were included in the study cohort. Characteristics of these patients are found in Table 1. The majority of the patients were female (93%), mean age was 32.1 years (SD: 12.4; range: 18–64), and the mean admission BMI and %IBW was 12.9 kg/m^2^ (SD: 1.6) and 61.6% (SD: 7.3), respectively. Subtypes of AN included 39 (53%) patients diagnosed with AN‐R, 28 (38%) patients diagnosed with AN‐BP, and 6 (8%) patients diagnosed with ARFID. Upon admission, patients received standard of care disease‐specific treatment. Nutritional support, guided by an registered dietitian was designed to start at 1400–1800 calories per day and increasing every 2–3 days based on weight trends. Daily electrolyte repletion was also performed as indicated, and spironolactone was used in purging patients to manage edema in secondary hyperaldosteronism.

**TABLE 1:** Patient characteristics

	Cohort *N* = 73	ETDQ‐7 score < 14.5 (*N* = 37)	ETDQ‐7 score ≥ 14.5 (N = 36)	Autophony symptoms and ETDQ‐7 score ≥ 14.5 (*N* = 16)
Female	68 (93%)	35	33	13
Male	5 (7%)	2	3	3
AN subtype				
AN‐R	39 (53%)	19	20	9
AN‐BP	28 (38%)	14	14	7
ARFID	6 (8%)	4	2	0

	Mean (SD)	Mean (SD)	Mean (SD)	Mean (SD)
Admit BMI	12.9 (1.6)	12.8 (1.3)	13.0 (1.8)	12.9 (1.5)
Admit %IBW	61.6 (7.3)	62.2 (8.3)	61.1 (6.2)	60.1 (6.4)
Age	32.0 (12.4)	30.6 (12.7)	33.5 (12.0)	32.1 (11.4)
ETDQ‐7 score	16.6 (8.9)	9.3 (2.0)	24.0 (7.1)	25.4 (7.9)

Abbreviations: AN, anorexia nervosa; AN‐BP, anorexia nervosa binge‐purge subtype; AN‐R, anorexia nervosa restricting subtype; ARFID, avoidant restrictive intake disorder; BMI, body mass index; ETDQ‐7, Eustachian Tube Dysfunction Patient Questionnaire; IBW, ideal body weight.

In the 73 patients enrolled, 36 (49%) patients scored 14.5 or higher on the ETDQ‐7 (Mean score: 3.4, SD: 1.0), indicating likely eustachian tube dysfunction. The 35 patients (44%) answered yes to one or more of the 3 autophony screening questions. Sixteen patients reported autophony and scored ≥14.5 on the ETDQ‐7. Statistical comparison of patients without autophony and those with clinically significant symptoms (reported autophony and ETDQ‐7 ≥ 14.5) can be seen in Table 2. No significant differences were found between the two groups.

**TABLE 2:** Comparison of symptomatic and asymptomatic patients

		Asymptomatic ETDQ‐7 < 14.5 (*N* = 37)	Symptomatic ETDQ‐7 ≥ 14.5 and reported autophony (*N* = 16)	
	Cohort *N* = 73	Mean (SD)	Mean (SD)	*t*‐test (DF)
Admit BMI (kg/m^2^)	12.9 (1.6)	12.8 (1.3)	12.9 (1.5)	0.15 (51) *p =* .88
Admit %IBW	61.6 (7.3)	62.2 (8.3)	60.1 (6.4)	0.88 (51) *p =* .38
Age (years)	32.0 (12.4)	30.6 (12.7)	32.1 (11.4)	−0.4 (51) *p =* .69
Kg/week gain	1.8 (0.7)	1.8 (0.8)	1.9 (0.5)	−0.34 (47.9) *p =* .73
DXA/lipodystrophy studies				
%Fat minus the head	14.5 (3.8)	14.2 (4.1)	13.6 (6.4)	0.35 (31) *p =* .73
%Fat total	15.3 (3.3)	14.9 (3.7)	14.5 (3.1)	0.34 (31) *p =* .74
Total fat mass (g)	5843.6 (1870.2)	5605.6 (2136.2)	5762.3 (1740.3)	−0.2 (31) *p =* .84
Total fat minus head (g)	4966.3 (1827.8)	4781.3 (20.92.0)	4864.2 (1723.7)	−0.11 (31) *p =* .91
Trunk limb fat ratio	1.1 (0.2)	1.1 (0.2)	1.3 (0.3)	−1.4 (31) *p =* .17
Laboratory values				
Prealbumin (mg/dl)	19.5 (7.1)	19.4 (7.4)	17.9 (6.1)	0.72 (50) *p =* .47

Abbreviations: BMI, body mass index; IBW, ideal body weight.

**FIGURE 1:**
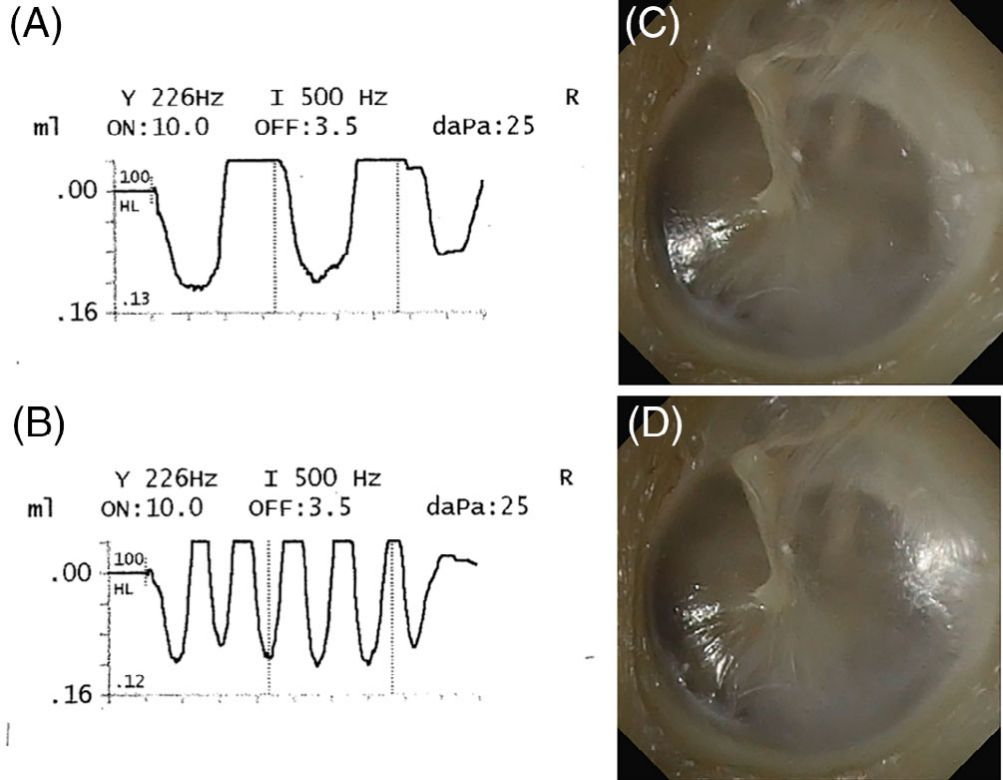
Objective evidence of patulous eustachian tube. Objective evidence of PET during testing of single subject is shown. Respiratory oscillations observed during reflex decay testing observed during (**A)** slow ipsilateral nasal breathing, and (**B)** fast ipsilateral nasal breathing. Tympanic membrane excursions visualized during video otoscopy: (**C)** inhalation, and (**D)** exhalation. Note the change in tympanic membrane bulges and the light reflex changes during exhalation.

**FIGURE 2:**
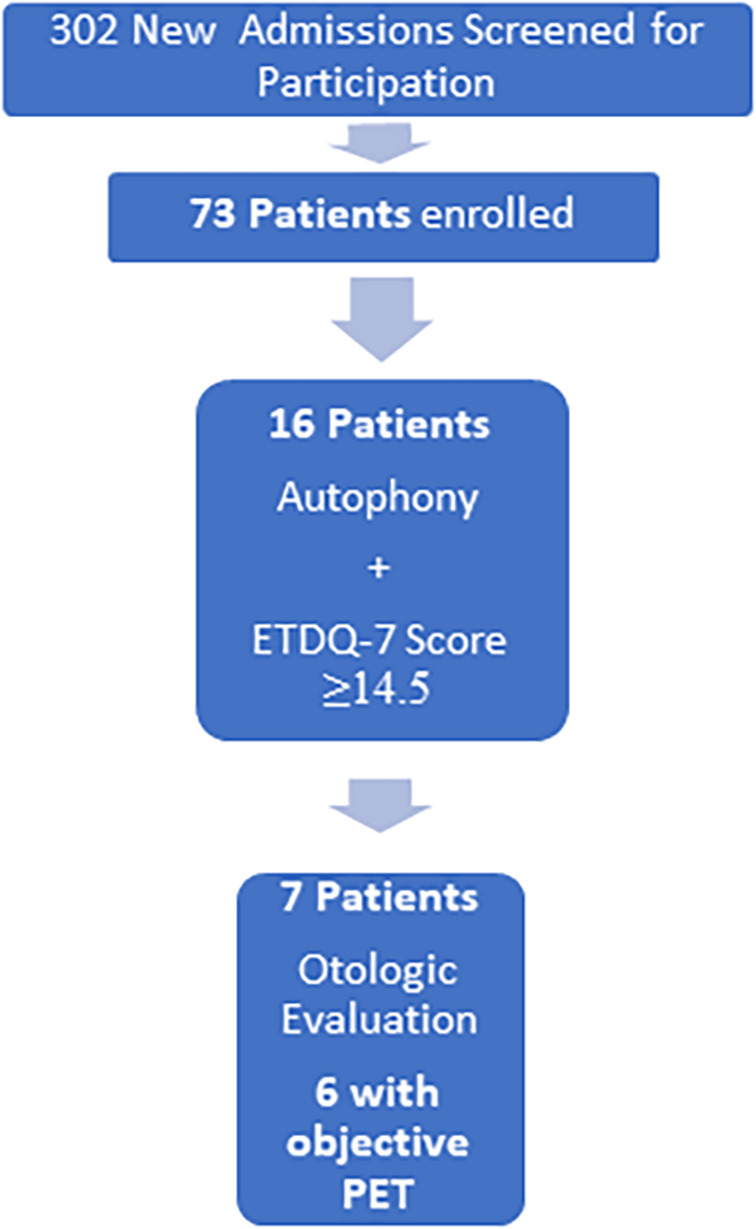
Study overview. Demonstration of overall cohort results is shown. This observational study revealed that at least six patients (8.2%) of the enrolled patients had patulous eustachian tube.

Of 16 patients with abnormal ETDQ‐7 scores who also reported autophony, seven patients completed the audiological and otolaryngology examinations. Four patients reported bilateral symptoms, and three patients reported unilateral symptoms. Six of the seven patients tested were found to have objective evidence of PET (Figure 1 **.)** The one patient with negative testing reported that their unilateral symptoms had resolved in the hours prior to the testing when an ipsilateral nasogastric tube had been placed. In all but one patient the results of the binocular otomicroscopy and reflex decay testing were the same. This single patient had positive TM excursions on binocular otomicroscopy during the evaluation by an otolaryngologist, but subsequently reported symptom resolution prior to audiological testing which occurred hours later. The reflex decay testing after symptom resolution confirmed the PET had resolved. A summary of the observational cohort and testing results can be seen in Figure 2.

Three patients also consented to have audiological testing repeated at a later date. Two patients were tested after they reported symptom resolution and had no objective findings of PET at that evaluation. A third patient was tested just prior to being transferred to another facility on 11th day of admission. Symptoms were improving and intermittent, but were present at the time of repeat testing. Continued respiratory oscillations during reflex decay confirmed patient still had PET.

In total, 10 objective evaluations were performed (20 ears). In every evaluation the objective findings of PET were present if the patient was having active symptoms in that ear at that time of the testing. In every asymptomatic ear, the findings were normal.

Every patient undergoing testing was found to have an otherwise normal head and neck exam (including ear exam) except cachexia. All patients had normal hearing on pure tone audiometry and word discrimination, normal tympanometry, and normal acoustic reflex screening. Only one patient had a history of prior ear conditions (pressure equalization tubes as a child.) Three of the seven had a history of allergic rhinitis, but none were taking nasal steroid sprays or oral decongestants. Of the seven patients undergoing objective testing voice autophony, breath autophony and aural pressure were reported in 7, 6, and 6 respectively. Pulsatile tinnitus, hyperacusis, and popping or cracking sounds were reported in 6, 5, and 5 respectively. Three patients noted exacerbation of symptoms with exercise, and five reported improvement with dependent head position. Improvement in symptoms with sniffing was reported in three. Six of the seven patients reported previous episodes of similar symptoms associated with weight loss. Two reported they have used autophony as an indication they should seek treatment for their weight loss. Three of the seven patients reported their symptoms were worst in the mornings‐ often resolving temporarily in the afternoon or evenings.

**FIGURE 3:**
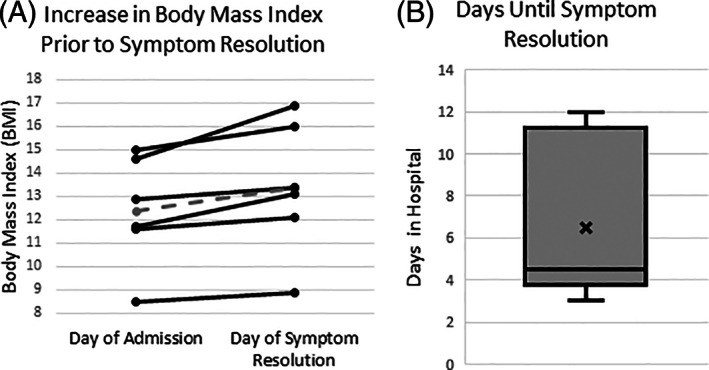
Resolution of autophony symptoms. Patients within the testing group were followed until autophony resolution. One patient still had symptoms on hospital day #11 when they transferred to another facility. In the remaining six patients, all improved their BMI prior to resolution of symptoms (A). Shown in the dotted line is the mean improvement from 12.4 to 13.4 kg/m^2^. (B) The cessation of autophony occurred rapidly. Number of days in the hospital prior to symptom resolution ranged from 3 days to 12 days (mean 6.5 ± 3.6).

Patients reported the autophony had been present from 2 weeks to 6 months in duration prior to admission (mean 10.4 weeks). After admission symptoms in all but one patient resolved within 12 days (the patient was still symptomatic when transferred to another facility.) Every patient gained weight prior to resolution of autophony (Figure 3.)

## 4 DISCUSSION

In this observational study of patients with severe eating disorders, PET was suspected based on questionnaires in 16/73 (22%) of patients and confirmed by PET testing in 6/7 patients that received the testing. The prevalence of PET in our cohort is likely greater given that 44% of patients reported autophony symptoms. However patients in other settings sometimes report aural symptoms when there are no objective findings of disease‐ and the rate of this reporting is higher in those with psychiatric diagnoses.^19^ We sought to identify clinically significant autophony with the addition of a validated tool to assess for eustachian tube dysfunction (ETDQ‐7).^15^ Although the ETDQ‐7 identifies individuals with either dilatory or patulous ET dysfunction, it is limited by being unable to discriminate between the two types.^13^, ^16^, ^17^ Moreover it does not include questions that address autophony. For this study we used an abnormal score on the ETDQ‐7 questionnaire (≥14.5), as well as positive responses to any of the three autophony‐specific screening questions to identify which patients had clinically significant symptoms.

Applying both of these criteria revealed that 22% of the cohort had significant autophony. We suspect nearly all of these patients have PET. Of the patients in this group who volunteered for objective testing, all but one was demonstrated to have PET. That patient had resolution of symptoms just prior to testing, after a nasogastric tube was placed on the same side as their aural symptoms. Overall 20 ear evaluations were performed revealing a 100% concordance between active autophony and objective evidence of PET. In every asymptomatic ear that was evaluated, the testing results were normal.

Clearly the significant autophony symptoms in our cohort are due to PET. This is the first study to confirm PET in patients admitted for treatment of severe AN, and the first study to demonstrate a specific subgroup of PET patients that experienced rapid resolution of symptoms with treatment. Patients reported the autophony had been present from 2 weeks to 6 months in duration prior to admission, but symptom resolution occurred for nearly all within 12 days. All patients experienced weight gain prior to symptom resolution, but with only a mean change in BMI from 12.4 to 13.4 kg/m^2^. This was particularly intriguing and raises the question whether mechanisms other than fat distribution are involved in the pathogenesis of PET. Our analysis revealed no differences between patients with clinically significant autophony and those without (Table 2 **)** including BMI, %IBW, pre‐albumin level at admission, and % body fat.

The rapid resolution along with the absence of symptoms in similarly malnourished patients suggest there are mechanisms involved beyond simply loss of body weight and fat distribution. Poe and Pyykko's study of eustachian tube openings was suggestive that hypertonia of the tensor veli palatini muscle may be an important factor.^20^ Perhaps the rapid resolution observed in this study represented a change in muscle tone. A deeper understanding of the pathogenesis in this unique patient population may produce insights into the mechanisms and possible treatment in other populations as well.

One major limitation to this study is the small number of objective evaluations that were completed. This was due to the limited patient population, volunteer nature of the evaluations, and the need for patient's to be medically stable to participate in the objective testing. Another challenge within our cohort was the report by several patients that symptoms followed a daily pattern of being most severe in the morning, and improving slowly throughout the day. This became a logistical barrier during our study as several patients had clinically significant autophony on screening but did not receive full objective evaluations because their symptoms were resolved when we discussed enrollment into the testing group later in the day. Another limitation of our analysis is the lack of a validated PET‐specific screening tool. There may have been patients with PET but who scored normally on the ETDQ‐7. Also there was no available data on the rate of weight loss in each cohort member prior to admission.

Despite these limitations, this study demonstrates that transient autophony in severe AN patients is due to PET, and that the underlying mechanisms are likely more complex than loss of body fat alone. Mucosal inflammation or edema may play a part in why some patients experience autophony while others do not, as was suggested by the participant who had symptom resolution shortly after a nasogastric tube placement. However many of the study patients reported symptoms were worst in the morning, when edema would be expected to mitigate symptoms in those spending the night supine. Further research will be needed to better understand the pathogenesis of PET in this patient population, why they achieve rapid symptom resolution, and how they differ from other populations with PET.

## 5 CONCLUSION

Our examination of 73 patients admitted for treatment of severe eating disorders demonstrated that PET was strongly suspected in 22% of the patients and a 100% concordance between active autophony and objective evidence of PET. Most of these patients experienced transient symptoms, resolving within days of disease‐specific treatment. Further study is warranted to understand the impact of PET in patients diagnosed with severe AN, and to gain insights into the pathophysiology of PET in all populations.

## FUNDING INFORMATION

Shana Glassman Memorial Endowed Chair in General Internal Medicine, University of Colorado School of Medicine.

## CONFLICTS OF INTEREST

No conflicts of interest to disclose.

## References

[1] O'Connor AF , Shea JJ . Autophony and the patulous eustachian tube. Laryngoscope. 1981;91(9 Pt 1):1427‐1435. doi:10.1288/00005537-198109000-00003 7346684

[2] Poe DS . Diagnosis and management of the patulous eustachian tube. Otol Neurotol. 2007;28(5):668‐677. doi:10.1097/mao.0b013e31804d4998 17534202

[3] Kobayashi T , Morita M , Yoshioka S , et al. Diagnostic criteria for patulous Eustachian tube: a proposal by the Japan Otological society. Auris Nasus Larynx. 2018;45(1):1‐5. doi:10.1016/j.anl.2017.09.017 29153260

[4] Yazici ZM , Gunes S , Koc RH , Gunes ME , Sayin İ . The impact of bariatric surgery on eustachian tube dysfunction. Eur Arch Otorhinolaryngol. 2021;278(3):689‐693. doi:10.1007/s00405-020-06128-y 32556787

[5] Muñoz D , Aedo C , Der C . Patulous eustachian tube in bariatric surgery patients. Otolaryngol Head Neck Surg. 2010;143(4):521‐524. doi:10.1016/j.otohns.2010.07.004 20869562

[6] Ward BK , Ashry Y , Poe DS . Patulous Eustachian tube dysfunction: patient demographics and comorbidities. Otol Neurotol. 2017;38(9):1362‐1369. doi:10.1097/mao.0000000000001543 28796094

[7] Young YH , Cheng PW , Ko JY . A 10‐year longitudinal study of tubal function in patients with nasopharyngeal carcinoma after irradiation. Arch Otolaryngol Head Neck Surg. 1997;123(9):945‐948. doi:10.1001/archotol.1997.01900090059008 9305244

[8] Plate S , Johnsen NJ , Nødskov Pedersen S , Thomsen KA . The frequency of patulous Eustachian tubes in pregnancy. Clin Otolaryngol Allied Sci. 1979;4(6):393‐400. doi:10.1111/j.1365-2273.1979.tb01771.x 527242

[9] Finsten RM , Faguet RA . Autophonia associated with an atypical eating disorder. J Clin Psychiatry. 1983;44(5):191. 6574128

[10] Karwautz A , Hafferl A , Ungar D , Sailer H . Patulous eustachian tube in a case of adolescent anorexia nervosa. Int J Eat Disord. 1999;25(3):353‐355. doi:10.1002/(sici)1098-108x(199904)25:3<353::aid-eat16>3.0.co;2-m 10192003

[11] Godbole M , Key A . Autophonia in anorexia nervosa. Int J Eat Disord. 2010;43(5):480‐482. doi:10.1002/eat.20702 19475655

[12] Hollis J , Mann S , Watters A , Oakes J , Mehler PS . Autophony in inpatients with anorexia nervosa or avoidant restrictive food intake disorder. Int J Eat Disord. 2022;55:388‐392. doi:10.1002/eat.23667 34993986

[13] Smith ME , Cochrane IL , Donnelly N , Axon PR , Tysome JR . The performance of patient‐reported outcome measures as diagnostic tools for Eustachian tube dysfunction. Otol Neurotol. 2018;39(9):1129‐1138. doi:10.1097/mao.0000000000001931 30106847

[14] APA . DIagnostic and Statistical Manual of Mental Disorders. 5th ed. APA; 2013.

[15] McCoul ED , Anand VK , Christos PJ . Validating the clinical assessment of eustachian tube dysfunction: the Eustachian tube dysfunction questionnaire (ETDQ‐7). Laryngoscope. 2012;122(5):1137‐1141. doi:10.1002/lary.23223 22374681 PMC3612400

[16] van Roeyen S , van de Heyning P , van Rompaey V . Value and discriminative power of the seven‐item Eustachian tube dysfunction questionnaire. Laryngoscope. 2015;125(11):2553‐2556. doi:10.1002/lary.25316 25891506

[17] Ikeda R , Kikuchi T , Miyazaki H , et al. The efficacy of the Eustachian tube dysfunction questionnaire (ETDQ‐7) for patulous Eustachian tube patient. Acta Otolaryngol. 2018;138(1):6‐9. doi:10.1080/00016489.2017.1366053 28880712

[18] Hamwi GJ . Therapy: Changing dietary concepts. In: Danowski TS , ed. Diabetes Mellitus: Diagnosis and Treatment. 5th ed. American Diabetes Association; 1964:73‐78.

[19] Okland TS , Gonzalez JR , Ferber AT , Mann SE . Association between patient review of systems score and somatization. JAMA Otolaryngol Head Neck Surg. 2017;143(9):870‐875. doi:10.1001/jamaoto.2017.0671 28617903 PMC5710288

[20] Poe DS , Pyykkö I . Measurements of Eustachian tube dilation by video endoscopy. Otol Neurotol. 2011;32(5):794‐798. doi:10.1097/MAO.0b013e31821c6355 21593699

